# Seasonal 25-hydroxyvitamin D levels in new childhood-onset SLE: associations with disease activity and peripheral lymphocyte subsets

**DOI:** 10.1136/lupus-2026-002044

**Published:** 2026-09-01

**Authors:** Chenxi Wei, Shufeng Zhi, Jiayin Li, Lijun Jiang, Xue Zhao, Qingxiao Su, Xingjie Qi, Hui Zhao, Zanhua Rong

**Affiliations:** 1 Department of Pediatrics, The Second Hospital of Hebei Medical University, Shijiazhuang, Hebei, China; 2 Key Laboratory of Rare Diseases of Hebei Province, Shijiazhuang, Hebei, China

**Keywords:** Systemic Lupus Erythematosus, Disease Activity, Pediatric, 25-hydroxyvitamin D, Peripheral lymphocyte subsets

## Abstract

**Objective:**

This study aimed to compare 25-hydroxyvitamin D (25-(OH)D) levels and peripheral lymphocyte subsets between children with new childhood-onset SLE and healthy controls across different seasons, and to explore the relationships of seasonal 25-(OH)D concentrations with the disease activity and peripheral lymphocyte subsets in paediatric patients with SLE.

**Methods:**

A retrospective study was conducted, enrolling 285 patients with new childhood-onset SLE and 175 healthy children. All participants were divided into a winter-spring group and a summer-autumn group according to sampling seasons. Serum 25-(OH)D levels and peripheral lymphocyte subsets were detected and quantified.

**Results:**

Compared with healthy controls, patients with SLE presented with significantly higher proportions of CD8^+^ T cells (p<0.05), alongside lower total lymphocyte counts, lower percentages of CD4^+^ T cells and Natural Killer(NK) cells, reduced CD4^+^/CD8^+^ ratio and decreased serum 25(OH)-D levels (all p<0.05). Within the SLE cohort, patients in the summer-autumn SLE group had markedly higher serum level of 25-(OH)D levels than those in the winter-spring group (18.1 vs 22.8 ng/mL; p<0.001). 25-(OH) D deficiency was correlated with a higher prevalence of lupus nephritis, higher Systemic Lupus Erythematosus Disease Activity Index 2000 (SLEDAI-2K) scores and lower serum calcium levels. In both seasonal subgroups, serum calcium and serum albumin showed weak positive correlations with serum 25-(OH)D levels. A moderate negative correlation was observed between SLEDAI-2K scores and 25-(OH)D levels in the winter-spring group, while only a weak negative correlation was detected in the summer-autumn group.

**Conclusions:**

Serum 25-(OH)D levels are affected by seasonal changes and closely correlated with disease activity in children with SLE. Patients with new childhood-onset SLE manifest abnormal peripheral lymphocyte subsets and insufficient 25-(OH)D.

**WHAT IS ALREADY KNOWN ON THIS TOPIC**

- Seasonal fluctuations can affect the 25-hydroxyvitamin D (25-(OH)D) levels in children, with higher concentrations observed in summer and autumn.
- 25-(OH)D is correlated with disease activity in SLE.
- However, relevant evidence is mainly derived from patients receiving long-term treatment, and studies focusing on paediatric populations remain scarce.

**WHAT THIS STUDY ADDS**

- Although several studies have explored the association between 25-(OH)D and SLE disease activity, few have further analysed its relationship with peripheral lymphocyte subsets.
- This study confirmed that children with new childhood-onset SLE in the Asian population have reduced 25-(OH)D levels and abnormal peripheral lymphocyte subsets.

**HOW THIS STUDY MIGHT AFFECT RESEARCH, PRACTICE OR POLICY**

- The 25-(OH)D levels in paediatric patients with SLE during summer–autumn correlated with disease activity, whereas no significant association was found between 25-(OH)D and peripheral lymphocyte subsets.
- Further investigations are required to elucidate the underlying mechanisms of 25-(OH)D in the pathogenesis of SLE.
- Limitations of this study include the exclusive enrolment of Asian participants; future research should include multiethnic cohorts to validate the findings.

## Introduction

 SLE is a chronic autoimmune disorder characterised by the production of diverse autoantibodies and multisystem involvement.^1^ The pathogenesis of SLE has not been fully elucidated. New childhood-onset SLE is prone to multiple organ complications, predominantly involving the renal (56.8%) and haematological (27.8%) systems.^2^

Vitamin D is an essential steroid hormone that regulates calcium and phosphorus homoeostasis. Accumulating evidence has demonstrated that vitamin D exerts extensive immunomodulatory effects on both innate and adaptive immune responses.^3–5^ Vitamin D is obtained via two major pathways: endogenous cutaneous synthesis stimulated by ultraviolet radiation, and exogenous intake from dietary sources and nutritional supplements.^6^ Serum 25-hydroxyvitamin D (25-(OH)D) is the most commonly used biomarker for evaluating vitamin D status.^7^ Due to variations in sunlight exposure, the 25-(OH)D levels are generally higher in summer and autumn than in winter and spring.^8^ Vitamin D deficiency is closely linked to the development of autoimmune diseases, and vitamin D supplementation has been proposed as a potential preventive or therapeutic strategy for such conditions.^3^ Multiple studies have reported an inverse correlation between serum 25-(OH)D levels and SLE disease activity.^9 10^

Extrafollicular T-B-cell interactions play a pivotal role in SLE pathogenesis.^11^ Among peripheral lymphocyte subsets (T cells, B cells and NK cells), T cells are most strongly associated with SLE progression, and a decreased CD4^+^/CD8^+^ ratio is a typical immunological feature of this disease.^12^

To exclude confounding effects of the clinical interventions, such as medications use and sunlight avoidance, and focus on the intrinsic characteristics of SLE itself, the present study exclusively enrolled patients with new childhood-onset SLE. Most existing studies on 25-(OH)D in SLE have focused on adult patients, while research regarding paediatric SLE is limited, and seasonal variations in 25-(OH)D levels in this population have not been systematically analysed. Accordingly, this study aimed to explore whether seasonal 25-(OH)D levels correlate with disease activity and peripheral lymphocyte subsets in children with new childhood-onset SLE.

## Methods

### Research subjects and data collection

This retrospective study was approved for a waiver of informed consent. A total of 285 children aged under 18 years newly diagnosed with SLE were recruited from the Department of Pediatrics Rheumatology Immunology and Nephrology, the Second Hospital of Hebei Medical University between November 2013 and November 2025. These patients were divided into the winter-spring group (December–May, n=119) and summer-autumn group (June–November, n=166) according to the season of enrolment. Meanwhile, 175 age-matched and gender-matched healthy children from the outpatient department were enrolled as the control group.

Study participants’ inclusion criteria were as follows: (1) Patients met either the 1997 SLE classification criteria revised by the American College of Rheumatology^13^ or the 2012 Systemic Lupus Erythematosus International Collaborating Clinics classification criteria for SLE;^14^ (2) Newly diagnosed patients with SLE; (3) Age younger than 18 years; (4) All the patients were ethnically Asian.

The exclusion criteria were as follows: (1) History of vitamin D supplementation or glucocorticoid therapy within the previous 3 months; (2) Presence of diseases interfering with vitamin D metabolism including diabetes, gastrointestinal surgery, inflammatory bowel disease and other chronic disorders.

Collected data included baseline characteristics (age, gender, body mass index), clinical manifestations and laboratory tests. Clinical manifestations comprised oral ulcers, non-scarring alopecia, cutaneous lesions, arthritis, lupus nephritis, serositis, vasculitis, pulmonary and neurological involvement. Laboratory examinations included routine blood tests, erythrocyte sedimentation rate, serum calcium, serum phosphorus, serum albumin (ALB), alkaline phosphatase (ALP), blood urea nitrogen, serum creatinine, complement 3, complement 4, antidouble-stranded DNA, Systemic Lupus Erythematosus Disease Activity Index 2000 (SLEDAI-2K) scores, peripheral lymphocyte subsets and serum 25-(OH)D levels.

### Measurement of serum 25-(OH)D levels

Serum 25-(OH)D was detected using a chemiluminescence immunoassay with commercial kits (Siemens Healthcare Diagnostics, USA), on an ADVIA Centaur XP automated analyser. Vitamin D status was classified based on serum 25-(OH)D concentrations: deficiency (≤20 ng/mL), insufficiency (20–30 ng/mL) and sufficiency (≥30 ng/mL).^15^ All analyses were performed separately by season.

### Detection of peripheral lymphocyte subsets

Flow cytometry was applied to identify peripheral lymphocyte subsets using surface markers CD16^+^56, CD19, CD3, CD4 and CD8 (BD Biosciences, USA). Samples were analysed on a NAVIOS flow cytometer (Beckman Coulter, USA). We quantified the proportions of CD16^+^CD56^+^NK cells, CD19^+^B cells, CD3^+^T cells, CD4^+^T cells and CD8^+^T cells, as well as the CD4^+^T/CD8^+^T ratio.

### Statistical analysis

All statistical analyses were performed using SPSS V.27.0 (IBM Corporation). Normally distributed continuous data were presented as mean±SD and compared using the independent-samples t-test or one-way analysis of variance. Non-normally distributed continuous data were expressed as median (IQR), and analysed via the Mann-Whitney U tests. Categorical data were described as cases (%), and compared using the χ^2^ test or Fisher’s exact test. Spearman’s correlation analysis was used to assess the relationships between 25-(OH)D levels and clinical indicators. A two-tailed value of p<0.05 was considered statistically significant.

## Results

No significant differences in demographic characteristics, clinical manifestations or most laboratory parameters were found between the two SLE groups, except for serum phosphorus levels, which were higher in the summer-spring group (online supplemental table 1).

For the healthy controls, the winter-spring subgroup included 77 girls (83.7%) with a median age of 12.3 (11.0–13.7) years; the summer-autumn subgroup included 68 girls (81.9%) with a median age of 11.9 (10.0–13.3) years. For patients with SLE: the winter-spring group had 99 girls (83.2%) aged 11.6 (9.7–13.3) years and the summer-autumn group had 138 girls (83.1%) aged 11.9 (10.0–13.3) years. In the winter-spring cohort, patients with SLE had a significantly higher proportion of CD8^+^ T cells (p<0.05), and lower percentages of CD4^+^T cells and NK cells, a reduced CD4^+^/CD8^+^ ratio, decreased total lymphocyte count and lower serum 25-(OH)D levels (p<0.05) compared with healthy controls. No intergroup differences in age, female proportion or CD19^+^B cells percentage were observed between the summer-autumn and winter-spring patient groups (table 1). In the summer-autumn cohort, patients exhibited obvious immunological abnormalities relative to healthy controls, increased proportions of CD8^+^T cells and CD19^+^B cells (both p<0.05), decreased total lymphocyte count, and reduced percentages of CD3^+^T cells, CD4^+^T cells and NK cells (all p<0.05). The CD4^+^/CD8^+^ ratio and serum 25-(OH)D levels were also significantly lower in patients with SLE (all p<0.05) (table 1).

**Table 1:** Comparison of 25-hydroxyvitamin D (25-(OH)D) levels and peripheral lymphocyte subsets in healthy controls and patients with SLE between the different seasons’ groups

	Winter-spring group (n%)			Summer-autumn group (n%)			
	Control (n=92)	SLE (n=119)	P value*	Control (n=83)	SLE (n=166)	P value†	P value‡
Age, M (P25–P75), years	12.3 (11.0–13.7)	11.6 (9.7–13.3)	0.081	11.3 (8.9–13.5)	11.9 (10.0–13.3)	0.307	0.452
Female, no. (%)	77 (83.7)	99 (83.2)	0.947	68 (81.9)	138 (83.1)	0.813	0.840
Lymphocyte, /μL (normal range 1500–4600)	2.7 (2.1–3.1)	1.3 (1.0–1.8)	**<0.001**	2.6 (2.0–3.4)	1.2 (0.9–1.8)	**<0.001**	0.738
CD3^+^T, M (P25–P75), (%)	72.3 (69.2–74.6)	71.3 (60.5–77.2)	0.395	73.6 (67.7–78.2)	68.8 (63.9–75.4)	**0.023**	0.806
CD4^+^T, M (P25–P75), (%)	37.4 (34.4–42.7)	31.2 (25.3–38.0)	**<0.001**	35.4±7.2	30.6±8.3	**0.003**	0.454
CD8^+^T, mean±SD, (%)	27.9±6.0	34.3±7.9	**<0.001**	30.4 (23.9–35.4)	33.0 (29.6–39.6)	**0.002**	0.610
CD4^+^T/CD8^+^T, M (P25–P75)	1.4 (1.1–1.6)	0.9 (0.7–1.2)	**<0.001**	1.3±0.5	0.9±0.3	**<0.001**	0.705
NK, M (P25–P75), (%)	8.3 (6.5–12.1)	6.4 (4.2–10.2)	**0.009**	9.2 (5.6–12.7)	6.1 (4.2–38.7)	**0.002**	0.517
CD19^+^B, M (P25–P75), (%)	16.8 (12.0–17.7)	17.7 (13.3–28.7)	0.055	15.8 (12.0–19.6)	20.4 (14.2–29.4)	**<0.001**	0.247
25-(OH)D, M (P25–P75), ng/mL	20.9 (15.8–29.7)	18.1 (14.0–22.8)	**0.003**	27.1 (22.4–34.4)	22.8 (18.1–27.6)	**<0.001**	**<0.001**

Bold values indicate significant p values.

Significant differences (p<0.05) are shown in bold. Student’s t-test, Wilcoxon rank-sum test or the χ2 test were used as appropriate.

* P value: Winter-spring controls group versus winter-spring SLE group.

† P value: Summer-autumn controls group versus summer-autumn SLE group.

‡ P value: Winter-spring SLE group versus summer-autumn SLE group.

NK, Natural Killer Cell.

**Figure 1:**
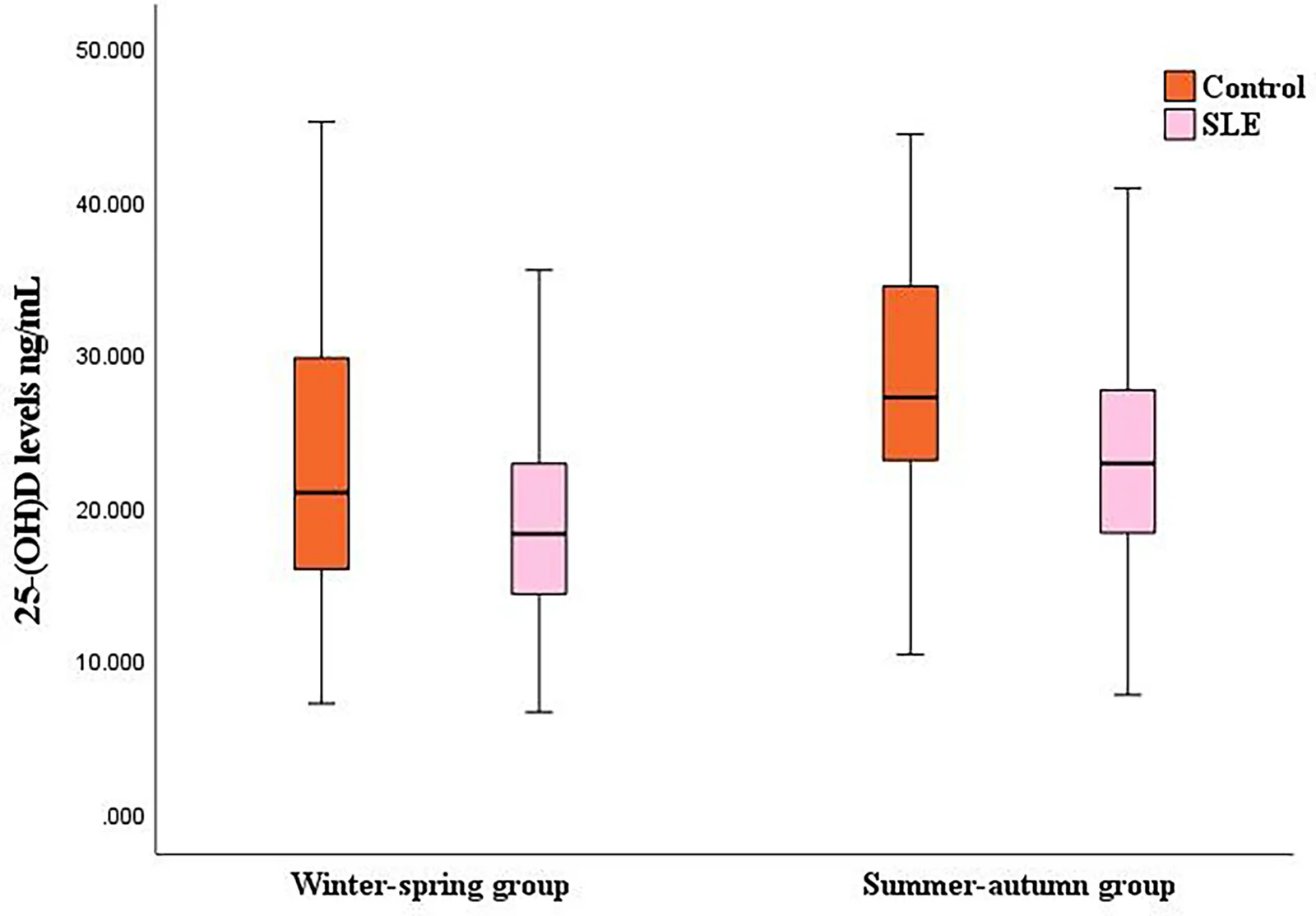
Comparison of seasonal 25-(OH)D levels in Healthy controls and patients with SLE. 25-(OH)D, 25-hydroxyvitamin D.

Within the overall SLE cohort, serum 25-(OH) D levels were significantly higher in the summer-autumn group than in the winter-spring group (p<0.05) (figure 1).

All patients with SLE were stratified into three subgroups according to their 25-(OH)D status for comparative analysis of clinical and laboratory indices. In the winter-spring group, patients with 25-(OH)D deficiency had a higher incidence of lupus nephritis and higher SLEDAI-2K scores (both p<0.05), as well as lower serum level of serum calcium levels (p<0.05), compared with those with sufficient vitamin D. In the summer-autumn group, patients with 25-(OH)D deficiency presented with significantly lower serum ALB and ALP levels than patients with adequate vitamin D (online supplemental table 2).

**Figure 2:**
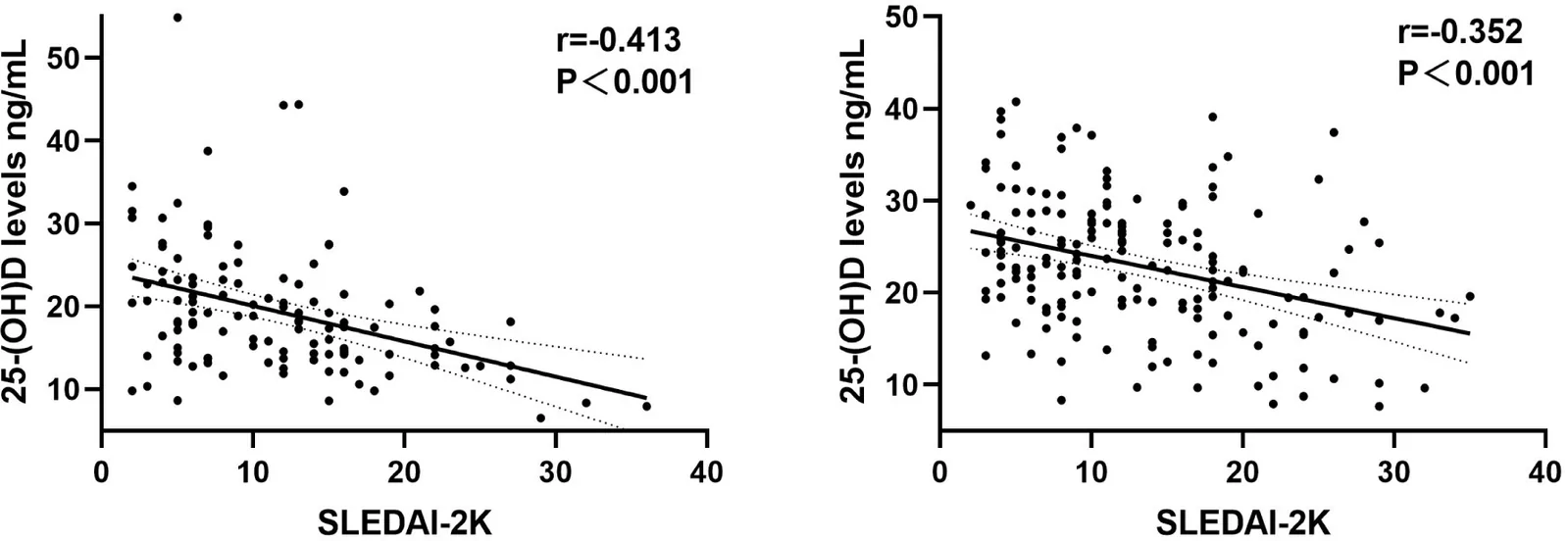
Associations of SLEDAI-2K with winter-spring 25-(OH)D level (left) and summer-autumn 25-(OH)D level (right) in patients with SLE. Spearman’s correlation analysis was used for statistical analyses. 25-(OH)D, 25-hydroxyvitamin D; SLEDAI-2K, Systemic Lupus Erythematosus Disease Activity Index 2000.

Correlations between serum 25-(OH) D levels and disease activity as well as clinical parameters are summarised in table 2. In the winter-spring group, 25-(OH) D levels were weakly positively correlated with serum calcium (r=0.246, p＝0.007) and ALB (r=0.205, p＝0.025), and moderately negatively correlated with SLEDAI-2K scores (r=−0.413, p<0.001) (figure 2). In the summer-autumn group, 25-(OH) D levels also showed weak positive correlations with serum calcium (r=0.359, p<0.001) and ALB (r=0.321, p<0.001), and a weak negative correlation with the SLEDAI-2K scores (r=−0.352, p<0.001) (figure 2). No significant correlations were detected between 25-(OH)D levels and peripheral lymphocyte subsets in paediatric patients with SLE (table 2).

**Table 2:** Correlation between 25-(OH)D levels and peripheral lymphocyte subsets/serological parameters/SLE disease activity markers

Disease activity markers	Winter-spring vitamin D level		Summer-autumn vitamin D level	
Correlation coefficient	P value	r	P value	r
Leucocyte	0.699	0.036	0.227	0.094
Lymphocyte	0.465	0.068	0.404	0.065
Haemoglobin	0.698	−0.036	0.052	0.151
Platelet	0.332	0.090	0.040	0.160
ESR	0.787	0.025	0.557	0.046
Serum calcium	**0.007**	**0.246**	**<0.001**	**0.359**
Serum phosphorus	0.169	0.127	0.210	0.098
ALB	**0.025**	**0.205**	**<0.001**	**0.321**
ALP	0.963	−0.004	0.011	0.198
SLEDAI-2K	**<0.001**	**−0.413**	**<0.001**	**−0.352**
CD3^+^T (%)	0.179	0.124	0.849	0.015
CD4^+^T (%)	0.347	0.087	0.716	0.028
CD8^+^T (%)	0.344	−0.087	0.508	−0.052
CD4^+^T/CD8^+^T	0.173	0.126	0.313	0.079
NK (%)	0.447	0.070	0.215	0.097
CD19^+^B (%)	0.263	−0.103	0.457	−0.058

Differences (p<0.05) are shown in bold. The thresholds for interpreting correlation strength: very strong (r>0.8), strong (r=0.6–0.8), moderate (r=0.4–0.6) and weak (r<0.4). Spearman’s correlation analysis was used for statistical analyses.

ALB, serum albumin; ALP, alkaline phosphatase; ESR, erythrocyte sedimentation rate; NK, Natural Killer Cell; 25-(OH)D, 25-hydroxyvitamin D; SLEDAI-2K, Systemic Lupus Erythematosus Disease Activity Index 2000.

## Discussion

SLE is a chronic autoimmune disease with multisystem involvement and highly variable clinical manifestations and complications. Accumulating evidence has confirmed that vitamin D plays a vital role in regulating immune responses.^9 10^ Nevertheless, studies exploring the association between vitamin D status and immunological profiles in paediatric SLE, especially those considering seasonal factors, remain limited. The present study enrolled treatment-naive children with new childhood-onset SLE and took seasonal variations in sunlight exposure (a key determinant of vitamin D synthesis) into account. We investigated the relationships of serum 25-(OH)D levels with disease activity and peripheral lymphocyte subsets in this population. Consistent with previous findings, our results revealed that children with SLE had higher CD8^+^T cell proportions, lower total lymphocyte counts, reduced percentages of, CD4^+^ T cells and NK cells, a decreased CD4^+^/CD8^+^ ratio and lower 25-(OH)D levels compared with healthy controls. Serum 25-(OH)D concentrations were markedly lower in the winter-spring group than in the summer-autumn group. In both subseasonal groups, serum calcium and ALB weakly correlated with 25-(OH)D levels. SLEDAI-2K scores were moderately negatively correlated with serum 25-(OH)D levels in the winter-spring group and weakly negatively correlated in the summer-autumn group, while no correlation was found between 25-(OH)D and peripheral lymphocyte subsets.

In the present study, lower vitamin D levels were observed in patients with SLE compared with healthy controls, a finding consistent with previous studies.^10 16^ Patients with 25-(OH)D deficiency had a higher risk of lupus nephritis, higher SLEDAI-2K scores and lower serum calcium levels. Serum 25-(OH)D levels were positively correlated with serum calcium and ALB, and inversely correlated with the SLEDAI-2K Score. The decreased serum calcium in patients with SLE may be explained by multiple mechanisms. First, renal impairment is common in Chinese children with new childhood-onset SLE, leading to proteinuria and hypoalbuminaemia, which further disrupt calcium-phosphorus metabolism; additionally, ALB interferes with calcium detection.^2 17^ Moreover, hypocalcaemia may exacerbate SLE activity by enhancing cellular immune activation.^18^ In the summer-autumn group, patients with 25-(OH)D deficiency had lower ALP levels. As a key biomarker of osteoblast activity and bone formation, reduced ALP indicates disturbed bone metabolism in patients with SLE.^19^

Pathologically, SLE is a characterised aberrant activation of autoreactive T cells and excessive autoantibody production by B cells. Anti-CD20 therapy can deplete peripheral B cells, inhibit abnormal T cell activation and differentiation, and induce long-term disease remission.^20^ In our study, patients with SLE in both seasonal groups had elevated CD8^+^ T cell proportions, reduced total lymphocyte counts, lower CD4^+^ T cell and NK cell percentages, and a decreased CD4^+^/CD8^+^ ratio. Patients in the summer-autumn group also had increased CD19^+^ B cells and decreased CD3^+^ T cells, yet these lymphocyte subset abnormalities were not associated with 25-(OH)D levels. Previous studies have reported that elevated B cell proportions combined with decreased T cell subsets (CD3^+^, CD3^+^/CD4^+^, CD3^+^/CD8^+^, CD3^+^/CD4^+^/CD8^+^ and CD3^+^/CD4^−^/CD8^−^) are correlated with co-infection in children with SLE.^21 22^ Therefore, detection of peripheral lymphocyte subsets helps clinicians evaluate infection risk and optimise treatment strategies for paediatric SLE.

The exact mechanisms by which vitamin D modulates SLE pathogenesis remain unclear. Antivitamin D antibodies detected in patients with SLE may neutralise the biological functions of vitamin D and participate in autoimmune processes.^23^ Vitamin D exerts anti-inflammatory effects via regulating multiple immune cells, including dendritic cells, macrophages, T cells and B cells, and also modulates autoantibody production in SLE.^24^ Vitamin D deficiency is linked to increased ANA expression and elevated proinflammatory cytokines such as Interferon-γ, IL-23 and IL-17.^25 26^ Animal experiments using MRL/lymphoproliferation mice have demonstrated that vitamin D suppresses the complement system proinflammatory cytokines (Tumor Necrosis Factor-α, IL-17 and Monocyte Chemoattractant Protein-1), and the Nuclear Factor-κB and Mitogen-Activated Protein Kinase signalling pathways.^27^

This study has several limitations. First, it is a single-centre retrospective study, which may introduce selection bias and information bias. Second, all participants were ethnically Asian, limiting the generalisability of the results. Third, we combined seasonal data for analysis to improve statistical power due to the sample size constraint; larger cohorts are required for stratified analysis in separate seasons. The strengths of this study include the exclusion of treatment and seasonal confounding factors, exclusive enrolment of patients with new childhood-onset SLE, and comprehensive analysis of disease activity, clinical manifestations and laboratory indices. Vitamin D deficiency is highly prevalent in SLE and contributes to autoimmune pathogenesis, making vitamin D supplementation a potential intervention for autoimmune diseases.^28^ However, real world studies have failed to confirm the efficacy of vitamin D supplementation in controlling SLE activity.^29^ Further research is urgently needed to clarify the molecular mechanisms of vitamin D in SLE.

## Conclusion

In children with new childhood-onset SLE, serum 25-(OH)D levels vary with seasons and are inversely associated with disease activity. These patients present with concurrent peripheral lymphocyte subset abnormalities and reduced serum 25-(OH)D levels. Vitamin D deficiency is correlated with a higher incidence of lupus nephritis, higher SLEDAI-2K scores and decreased serum calcium. Lower 25-(OH)D levels are also accompanied by reduced serum calcium and ALB. Regular monitoring of vitamin D status is recommended for the clinical management of paediatric patients with SLE.

## Supplementary material

10.1136/lupus-2026-002044 online supplemental file 1

## Data Availability

Data are available upon reasonable request. All data relevant to the study are included in the article or uploaded as supplementary information.
