# Supplementary material for: Seasonal 25-hydroxyvitamin D levels in new childhood-onset SLE: associations with disease activity and peripheral lymphocyte subsets
Source: Lupus Sci Med. 2026 Sep 1;13(2):e002044. doi: 10.1136/lupus-2026-002044 (PMC13536044; doi:10.1136/lupus-2026-002044)
Supplement: Supplementary file 1 [file lupus-13-2-s001.pdf]

Supplemental table 1. Demographic, clinical and laboratory characteristics of children patients with SLE

Features	Winter-spring group(n=119)	Summer-autumn group(n=166)	p-Value
Age,M (P25 ,P75),years	11.6 (9.7,13.3)	11.9 (10.0,13.3)	0.452
Female,no.(%)	99 (83.2)	138 (83.1)	0.840
BMI,M (P25 ,P75),kg/m^2	17.0 (15.3,19.5)	18.0 (15.6,20.0)	0.153
Oral ulcers,no.(%)	12 (10.1)	14 (8.4)	0.633
Non-scarring alopecia,no.(%)	12 (10.1)	14 (8.4)	0.633
Skin rash,no.(%)	63 (52.9)	93 (56.0)	0.606
Arthritis,no.(%)	21 (17.6)	33 (19.9)	0.635
Nephritis,no.(%)	44 (37.0)	71 (42.8)	0.325
Serositis,no.(%)	22 (18.5)	40 (24.1)	0.258
Vasculitis,no.(%)	6 (5.0)	7 (4.2)	0.742
Pulmonary,no.(%)	8 (6.7)	17 (10.2)	0.300
Neurologic,no.(%)	16 (13.4)	23 (13.9)	0.921
Leukopenia,M (P25 ,P75),×10^3/μL(normal range 4.3-11.3)	3.9 (3.0,5.6)	3.7 (2.7,5.8)	0.266
Hemoglobin,M (P25 ,P75),g/L(normal range 110-156)	107.0 (90.0,119.0)	108.5 (88.8,121.0)	0.521
Platelets,mean±SD,×10^3/μL(normal range 167-453)	173.4±113.23	155.8±99.4	0.166
ESR, M (P25 ,P75),mm/h (normal range 0－25)	37.0 (22.0,65.0)	41.0 (22.0,65.0)	0.870
Serum calcium,M (P25 ,P75),mmol/L (normal range 2.1–2.8)	2.2 (2.0,2.3)	2.1 (2.0,2.3)	0.599
Serum phosphorus,M (P25 ,P75),mmol/L(normal range 1.3–1.9)	1.4 (1.2,1.6)	1.5 (1.3,1.7)	**0.030**
ALB,M (P25 ,P75),g/L(normal range 39–54)	36.1 (30.8,41.6)	35.4 (30.0,41.0)	0.427
ALP,M (P25 ,P75),U/L(normal range 63–327)	102.0 (74.0,138.0)	99.5 (75.8,150.5)	0.677
BUN,M (P25 ,P75),mmol/L(normal range 2.5–6.5)	4.1 (3.3,5.6)	4.3 (3.4,5.8)	0.455
SCr,M (P25 ,P75), μmol/L(normal range 27–66)	44.0 (38.0,49.6)	45.4 (46.0,59.0)	0.062
Low levels of C3,no.(%)	104 (87.4)	144 (86.7)	0.759
Low levels of C4,no.(%)	93 (78.2)	135 (81.3)	0.595
Anti-dsDNA positivity,no.(%)	84 (70.6)	122 (73.5)	0.589
SLEDAI-2K, M (P25 ,P75)	11.0 (6.0,16.0)	11.5 (7.0,18.0)	0.148

Note: Abbreviations: SLE, systemic lupus erythematosus; BMI, body mass index; ESR,erythrocyte sedimentation rate; ALB, serum albumin; ALP, alkaline phosphatase; BUN, blood urine nitrogen; SCr, serum creatinine; C3, complement 3; C4, complement 4; Anti-ds DNA, anti-double-stranded DNA; SLEDAI-2K, systemic lupus erythematosus disease activity index 2000; M, Median; P, Quartile. **Significant differences (p ＜0.05) are shown in bold. Student's t-test, Wilcoxon Rank-Sum test, or the chi-square test were used as appropriate.**

Supplemental table 2. Comparison of seasonal 25-(OH)D levels and clinical manifestations of SLE.

	Winter-spring group,n(%)			
	25-(OH)D sufficiency group(n = 10)	25-(OH)D insufficiency group(n = 36)	25-(OH)D deficiency group (n = 73)	p-Value
Oral ulcers	0(0)	5(13.9)	7 (9.9)	0.264
non-scarring alopecia	1 (10.0)	4 (11.1)	7 (9.9)	0.970
Skin rash	4 (40.0)	18 (50.0)	41 (56.2)	0.576
Arthritis	1 (10.0)	6 (16.7)	14 (19.2)	0.740
Nephritis	2 (20.0)	7 (19.4)	35 (47.9)	**0.006**
Serositis	0 (0)	6 (16.7)	16 (21.9)	0.095
ESR, mm/h (normal range 0﹣25)	25.5 (10.8,77.0)	48.5 (28.0,72.0)	34.0 (20.5,59.5)	0.239
Serum calcium,mmol/L (normal range 2.1–2.8)	2.2 (2.1,2.4)	2.2 (2.1,2.3)	2.1 (2.0,2.3)	**0.013**
Serum phosphorus,mmol/L(normal range 1.3–1.9)	1.5±0.3	1.4±0.3	1.4±0.3	0.683
ALB,g/L,(normal range 39–54)	39.2±7.7	37.3±6.8	33.4±8.9	0.062
ALP,U/L,(normal range 63–327)	83.5 (74.3,187.8)	102.0 (74.5,137.5)	102.0 (72.1,135.0)	0.887
Anti-dsDNA positivity,no.(%)	6(60.0)	21 (58.3)	57 (78.1)	0.080
SLEDAI-2K, mean (SD)	5.0 (2.0,12.3)	7.5 (5.0,12.0)	14.0 (7.0,17.5)	**<0.001**
	Summer-autumn group,n(%)			
	25-(OH)D sufficiency group(n = 27)	25-(OH)D insufficiency group(n =78)	25-(OH)D deficiency group (n = 61)	p-Value
Oral ulcers	1 (3.7)	6 (7.7)	7 (11.5)	0.429
non-scarring alopecia	2 (7.4)	5 (6.4)	7 (11.5)	0.563
Skin rash	16 (59.3)	41 (52.6)	36 (59.0)	0.699
Arthritis	6 (22.2)	14 (17.9)	13 (21.3)	0.838
Nephritis	8 (29.6)	29 (37.2)	34 (55.7)	**0.029**
Serositis	6 (22.2)	14 (17.9)	20 (32.8)	0.124
ESR, mm/h (normal range 0﹣25)	46.0 (22.0,68.0)	38.0 (21.8,69.3)	42.0 (22.0,61.5)	0.924
Serum calcium,mmol/L (normal range 2.1–2.8)	2.3 (2.1,2.4)	2.2 (2.1,2.3)	2.1 (1.9,2.2)	**<0.001**
Serum phosphorus,mmol/L(normal range 1.3–1.9)	1.6 (1.3,1.7)	1.5 (1.4,1.7)	1.5 (1.2,1.6)	0.276
ALB,g/L,(normal range 39–54)	37.1 (34.0,43.3)	37.6 (30.1,41.3)	32.7 (20.9,37.8)	**<0.001**
ALP,U/L,(normal range 63–327)	112.0 (93.0,153.0)	111.2 (85.0,175.3)	85.0 (62.0,117.0)	**0.005**
Anti-dsDNA positivity,no.(%)	21(77.8)	51(65.4)	50 (82.0)	0.077
SLEDAI-2K, mean (SD)	8.0 (5.0,18.0)	10.0 (6.0,15.0)	17.0 (9.0,23.5)	**<0.001**

Note: Abbreviations: 25-(OH)D, 25-hydroxyvitamin D; SLE, systemic lupus erythematosus; ESR,erythrocyte sedimentation rate; ALB, serum albumin; ALP, alkaline phosphatase; Anti-ds DNA, anti-double-stranded DNA; SLEDAI-2K, systemic lupus erythematosus disease activity index 2000. **Significant differences (p<0.05) are shown in bold. Student's t-test, Wilcoxon Rank-Sum test, analysis of Variance or the chi-square test were used as appropriate.**
